# Optimizing Healthcare Expenditure for Spinal Cord Stimulation in Italy: The Value of Battery Longevity Improvement and a Direct-to-Implant Approach

**DOI:** 10.36469/001c.116177

**Published:** 2024-05-28

**Authors:** Federica Tito, Gianfranco Sindaco, Simon Eggington, Elisa Tacconi, Francesca Borghetti, Mara Corbo, Gilberto Pari

**Affiliations:** 1 Medtronic Italia S.p. A., Milan, Italy; 2 Pain Unit Santa Maria Maddalena Hospital, Occhiobello, Italy; 3 Medtronic International Trading Sàrl, Tolochenaz, Switzerland; 4 Medtronic Italia S.p. A, Milan, Italy; 5 Medtronic Italia S.p. A, Milan, Italyv

**Keywords:** spinal cord stimulation, direct-to-implant, economic analysis, PC battery longevity, cost-saving

## Abstract

**Background:** Spinal cord stimulation (SCS) is a treatment for chronic intractable pain powered by an implantable pulse generator that may be rechargeable or not rechargeable (NR). It is performed in 2 stages (a trialing phase followed by permanent device implantation) and necessitates 2 hospitalizations, which may increase infection risk. **Objective:** This analysis explores the cost impact of improvements in battery longevity and the adoption of 1-step (direct-to-implant [DTI]) SCS implantation. **Methods:** Since 2019, 3 leading NR-SCS devices have been launched: Device A (2019), Device B (2020), and Device C (2021). The battery longevity of the newest Device C was estimated at comparable stimulation settings for Devices A and B. A Markov model simulated individual patient pathways across 2 scenarios: Device A vs Device C and Device B vs Device C (both with the DTI approach and 2-step approach). Costs considered were the initial device implantation procedure, device replacements, and serious adverse event (SAE) management. Italian diagnosis-related group (DRG) tariffs were applied for costs, and a 15-year time horizon was used. **Results:** Over 15 years, using a DTI approach, the undiscounted total costs for Device A vs Device C were €26 860 and €22 633, respectively, and €25 111 and €22 399 for Device B vs Device C, respectively. Compared with Devices A and B, Device C offered savings of €4227 and €2712, respectively; similar savings were predicted with a 2-step implant approach. **Discussion:** The battery longevity of NR-SCS devices directly impacts long-term costs to a payer. The longer the device lasts, the lower mean total cumulative costs the patient will have, especially with regard to device replacement costs. With novel devices and specific programming settings, the lifetime cost per patient to a payer can be decreased without compromising the patient’s safety and positive clinical outcome. **Conclusions:** Extended SCS battery longevity can translate into tangible cost savings for payers. The DTI approach for SCS supports National Healthcare System cost efficiencies and offers the additional benefits of optimizing operating room time while having only one recovery period for the patient.

## BACKGROUND

Neuropathic pain is a complex and disabling condition that affects 7% to 10% of the adult population in Europe and the Americas^1,2^ and substantially impacts health-related quality of life.^3,4^ In particular, the prevalence of chronic low back pain is approximately 19.6% in the population^5^ aged between 20 and 59 years. Neuropathic back and leg pain can often be successfully treated, but the associated back pain component, which may have both neuropathic and nociceptive etiologies, can be more difficult to treat. Up to 50% of patients with neuropathic pain fail to obtain pain relief from analgesic medication.^6^

Spinal cord stimulation (SCS) is a treatment for chronic intractable pain powered by an implantable pulse generator with a rechargeable (RC) or nonrechargeable (NR) battery. Recent studies have challenged the earlier dogma of increased longevity with RC systems by showing that NR systems were the most cost-effective option.^7^ Real-world analysis of Medicare claims data demonstrated that the clinical longevity of neurostimulator devices is similar for NR and RC batteries.^7^

Spinal cord stimulation is mainly applied in failed back surgery syndrome (FBSS), neuropathic, peripheral ischemic, amputation, and visceral pain^8,9^ ; and disorders involving long-term chronic pain.

The decades-long use of SCS for treating a variety of pain syndromes has been validated by multiple randomized trials that confirm the benefit of SCS in well-selected patients compared with traditional medical management.^10^ SCS is a cost-effective clinical treatment with better long-term outcomes at lower lifetime healthcare costs in such patients.^11^

SCS is generally performed in 2 stages: the trialing phase, followed by permanent device implantation. The first trialing phase allows different evaluations to be made; from the patient’s perspective, it gives the opportunity to evaluate the pain relief and assess the electrical current consumption from the device, which may impact the choice of the pulse generator to be implanted, as reported by Chincholkar et al.^12^ However, as reported by Duarte and Thomson,^13^ screening trials imply higher healthcare resource consumption, mainly due to duplication of procedures and hospitalization. The recommendation that all candidates for SCS should undergo a screening trial before permanent SCS implantation is largely based on expert opinion rather than firm evidence.^14^ Moreover, prolonged home SCS screening trials expose patients to a higher risk of infection.^15^

A cause-effect relationship between trial duration and the risk of infection has been well demonstrated in the PROMISE RCT study, although, for simplicity, this aspect has not been considered in the model.^15^ Nevertheless, treatment guidelines propose that under appropriate infection control conditions, the staged trial and completion implant pathway can be utilized in selected patients without a significant increase in infection rates.”^16^ For any patient, developing an infection during SCS treatment is a significant event that can obviate any therapeutic benefit provided by SCS, while the infection that manifests only after implantation of a complete system, precipitating its removal and possible replacement, also substantially increases the cost of the therapy.^15^

Technological advances and an increased understanding of the therapy area have resulted in better and more reliable SCS treatments. After surgery, device settings are individually programmed to achieve optimal pain relief; as a result, device settings vary between patients. Improvements in battery technology have allowed the development of NR extended-life primary-cell devices. This kind of device decreases the number of replacements as well as the possible complications related to the intervention.

This analysis explores the cost impact of both improvements in battery longevity and the adoption of a 1-step (direct-to-implant [DTI]) SCS approach within the Italian National Health System (NHS).

## METHODS

The model was developed to calculate the economic value of the Vanta^™^ Primary Cell (PC) device manufactured by Medtronic (Device C), compared with the Proclaim™ XR PC device manufactured by Abbott (Device A), and Alpha™ PC manufactured by Boston Scientific (Device B), based on improvements in longevity and related costs avoided from less frequent device replacements.^17,18^

Patients with chronic back and leg pain were the focus of the model, inclusive of FBSS and complex regional pain syndrome. The model’s perspective was the NHS, and the time horizon was 15 years.^19^ A DTI approach was modeled such that patients would receive implants directly with the full device kit as opposed to having 2 separate procedures (a test implant to determine patient response, followed by a permanent implant of the full device kit).

Economic models can use a patient-level or cohort-level modeling approach to estimate the expected costs and outcomes across a particular population. This model adopted a patient-level simulation.^20^

A Markov microsimulation approach was used to count the number of device replacements and adverse events per patient with a monthly cycle length. The events modeled included battery replacement, permanent device explantation, short-term and long-term adverse events, and patient mortality. To ensure the stabilization of the results, each model run was based on 20 000 patients. The model was developed in Microsoft Excel with Visual Basic for Applications.

Data inputs were taken from published literature, supplemented by data from the Medtronic Product Surveillance Registry and additional analyses from the French spinal cord stimulation registry.^21^

### Model Structure

The model contained 3 health states:

- **On therapy (post-system implant/system replacement):** Patients began the model in this health state and could also re-enter the state if they suffered an SAE requiring full system replacement. Patients remained in this state until they (1) had an SAE requiring device replacement or a device replacement due to battery depletion, (2) withdrew from therapy, or (3) died.
- **Off therapy**: Patients entered this state in the case of permanent explantation (removal) of the device kit (for any reason). Once off therapy, it was assumed that patients would not receive another device.
- Dead

**Figure 1.**
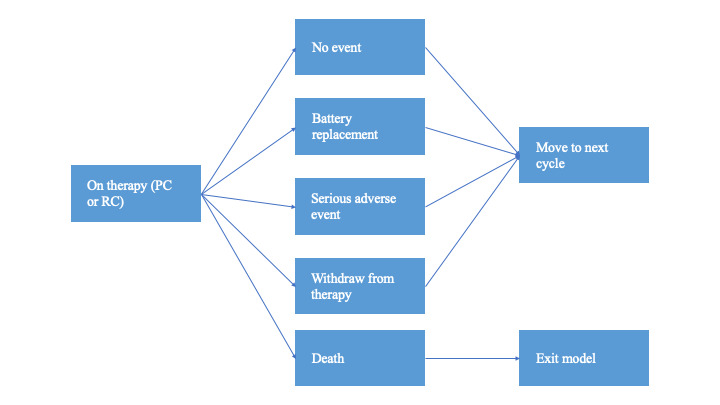
Possible Events in Each Monthly Cycle

Within any model cycle (duration, 1 month), patients could have an implantable pulse generator (IPG) replacement, experience an SAE, die, or have none of these events (**Figure 1**). If the patient had an SAE, the event was assumed to be resolved within the current cycle (withdrawal from therapy was modeled as a separate event to capture withdrawals related to SAEs or for any other reason). Each patient could have a maximum of 1 event per cycle (ie, they could not have a battery replacement and an adverse event in the same cycle).

### Treatment Groups

Three different devices (Device A, Device B, and Device C) were included in the model, allowing different comparisons to be made (the model did not include rechargeable devices). The model made 2 separate comparisons: Device C vs Device A and Device C vs Device B. This approach was taken due to the different device settings reported by Abbott and Boston Scientific for their respective devices. For each comparison, the longevity of Device C was recalculated using the different device settings for Device A and Device B; thus, a 3-way comparison of the devices was not possible. In each group, patients received an implanted device from the specified manufacturer at the start of the model and received an equivalent device for the remainder of the model at the time of planned IPG replacement or when a replacement device was needed due to an SAE. Thus, the model did not allow patients to switch between different manufacturers.

### Model Outcomes

The model recorded several pieces of data to demonstrate the economic value of the NR device. In all cases, the numbers were based on the average results across the 20 000 patients run through the model for each treatment group:

- Total costs (undiscounted) split into 4 categories:
  1. Initial device kit and its implantation
  2. Battery replacements (device and procedure costs)
  3. SAE management
  4. Device explant costs (for patients who withdraw from therapy)
- Mean number of battery replacements per patient
- Mean number of SAEs per patient
- Mean survival time per patient
- Proportion of patients withdrawing from therapy

### Data Inputs

**Patient demographics:** Based on data from the PROCESS RCT, patients were assumed to have a mean age of 50.39 years at baseline. The proportion of male patients was also taken from the PROCESS study,^22^ which reported a value of 51%. Patients were assumed to have the same mortality risk as a gender- and age-matched general population in Italy; thus, no disease-specific mortality risk was added. This is consistent with the approach used in a previous cost-effectiveness analysis of spinal cord stimulation for patients with FBSS.^19^ Italian life tables were used to derive monthly mortality probabilities by age and gender.^23^

**Serious adverse event incidence**: The model incorporated serious adverse events (SAEs) only, to focus on events having a significant economic impact. The risk of device-related SAEs was divided into short-term and long-term risks to distinguish between procedure-related events and those that were either device- or disease-related. Data on the rate of SAEs were taken from the French SCS registry,^21^ in which 89 patients (22%) had at least 1 SAE during a mean follow-up of 2 years. The events modeled were divided into 3 time periods according to time since the most recent device implant/ replacement:

- Within 30 days of device implant/replacement
- Between 30 and 180 days of device implant/replacement
- More than 180 days after device implant/replacement

This time-dependent element was incorporated to reflect the higher event rates, generally observed shortly after the implantation or replacement of a device. **Table 1** shows the probabilities used in the model for each time period.

**Table 1.** Serious Adverse Event Incidence (Monthly Probabilities)

Time Since Implantation/ Replacement (days)	Monthly Probability (%)
≤30	2.49
31-180	1.3
>180	1.1

**Adverse event management:** Management of SAEs was also largely based on data from the French registry, and the clinical authors of this paper (G.P., G.S.) confirmed that these strategies are consistent with practice in their centers in Italy, allowing hospitalization and surgery costs to be applied appropriately. The most commonly reported SAEs in the French registry were lead fracture, persistent pain despite stimulation, lead migration, and infection. This analysis considered subgroups of events with a cost applied to each event group to reflect the severity and the need for surgical intervention and/or the replacement of the device kit. The first split of events concerned whether the patient was hospitalized due to the SAE. Brinzeu et al^21^ reported that of the 89 patients with at least 1 SAE, 84 patients (94.4%) were hospitalized. Of these 84 patients, 64 (76.2%) required surgery; the remaining 20 hospitalized patients (23.8%) were therefore assumed not to have had surgery.

**Figure 2.**
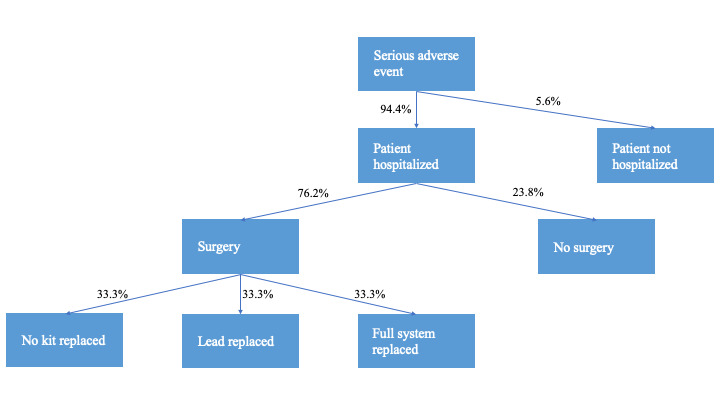
Patient Pathways Following a Serious Adverse Event

Among patients requiring surgery, 3 outcomes were considered: surgery with no device kit replacement; surgery with lead replacement; and surgery with full system replacement. Data are lacking concerning the proportion of patients managed in each way (and management varies according to event type); for this reason, one-third of patients requiring surgery were assumed to fall into each of these categories (**Figure 2**). In all cases, the procedure(s) needed for SAE management were assumed to occur in the same cycle as the event itself. The cost associated with each pathway is described in detail below.

**Device longevity:** Device longevity was fixed according to which device each patient was assigned to in the model. As previously described, the model was designed to make 2 comparisons (Device C vs Device A and Device C vs Device B), with different longevity assumptions made for each device based on stimulation settings published by Abbott and Boston Scientific, in their corresponding device product manuals.^17,18^ The longevity of these devices was evaluated according to the reported procedures, and the device settings data were entered into the longevity estimator for Device C. The estimator utilizes the current stimulation settings to estimate how long Device C would last under different scenarios, specifically by considering the settings of competitor devices. The results are presented in **Table 2**, which displays the average longevity of each device as implemented in the model. Notably, for Device C, two longevity values are provided: one using the settings from Device A and another using the settings from Device B.

**Table 2.** Mean Device Longevity by Device Manufacturer

**Comparator Device**	**Device Settings Assumed**	**Comparator Longevity (Years)**	**Vanta™ PC Longevity (Years)a**
Abbott Proclaim™ XR PC	Frequency: 50 Hz	4.5	8.31
	Pulse width: 225 μs		
	Amplitude: 5 mÅ		
	Impedance: 500 Ω		
Boston Scientific Alpha™ PC	Frequency: 40 Hz	5.1	9.27
	Pulse width: 280 μs		
	Amplitude: 4.1 mÅ		
	Impedance: 730 Ω		
	No. areas: 1		
	No. contacts: 2		

^a^Calculated based on device settings from the comparator device.

These longevity estimates were applied to both initial and replacement devices, such that no decline in longevity was modeled for patients having replacement devices. Patients would continue to have their devices replaced up to the end of the model horizon or until death, whichever occurred first.

At the time of device replacement due to battery exhaustion, the model also incorporated the option to replace the device leads on the basis that a surgical procedure is already being performed and occasional lead replacements are necessary. Based on Italian clinical practice, it has been assumed that 3% of battery replacements would also involve the replacement of both leads. This parameter was applied to all 3 device types.

**Patient withdrawal:** The model incorporated an ongoing risk of treatment withdrawal due to inadequate pain relief but also permanently because of SAEs. The monthly probability of permanent withdrawal was set to 0.35%, derived from a reported rate of 8% over 24 months in the French SCS registry.^21^ These data were consistent with other treatment withdrawal rates found in the literature. For example, Van Buyten et al^24^ reported a 19% withdrawal rate at 5 years due to inadequate pain relief, Nissen et al^25^ reported a long-term outcome of spinal cord stimulation in failed back surgery, with 25% withdrawal over a 6-year follow-up period from a single center in Finland.

**Cost**: A national payer perspective was adopted for the calculations. National DRG tariffs were used to determine the cost of implantation, replacement, and explantation as well as the management of SAEs, and the costs of the device kit were assumed to be included in the relevant tariffs (for this reason, no difference in device price between manufacturers was modeled). Costs of drug therapy, ongoing follow-up, and other alternative treatment options (eg, physiotherapy) were not considered in this analysis. Thus, the cost results produced by the model should not be used as a proxy for the overall disease management costs of patients with FBSS or complex regional pain syndrome.

The model was designed to record and output costs covering 4 main categories:

- Cost of implantation
- Cost of device replacements (due to battery depletion)
- Cost of managing SAEs
- Cost of permanent device explantation (due to withdrawal).

The Italian DRG tariffs were applied for the cost of a pre-operative hospitalization and for the device implantation and replacement procedures (DRG Manual ICD-9-CM-2007 Version 24.0. Max National DRG Tariffs 2012). Then, as a DTI approach was modeled, the cost of 1 hospitalization for each patient was avoided. Cost data from 2012 are still valid in the current year of analyses (2024), and no conversion was required.

Costs were not discounted in the base-case analysis, in line with recommendations for budget impact analyses,^26^ but discounted results were calculated as an additional scenario for completion. A full list of the unit costs used in the model is provided in **Table 3**, together with the source and any comments.

**Table 3.** Unit Costs Used in the Model

**Cost Component**	**Unit Cost (€)**	**Source**	**Comments**
Device implantation	8973	€8413 * 91% (DRG 532a) + €14 639 *	Weighted cost of DRG tariffs with (€14 639) and
procedure (all device types),		9% (DRG 531a)	without (€8413) complications. Includes costs of
including device kit cost		% from report SDO 2020b	device and kit.
Battery replacement	3409	€2326 * 87% (DRG 8a) + €10 658 *	Weighted cost of DRG tariffs with (€10 658) and
procedure (all device types),		13% (DRG 7a)	without (€2326) complications. Includes costs of
including device cost		% from report SDO 2020b	device.
SAE requiring full system	16 826	€8413 * 2 – DRG 532a	Explant + new implant procedure. The DRG without
explantation and re-			CC was applied in both hospitalizations for 8.65% of
implantation (infection)			SAEs which were infections.
SAE requiring full system	8413	€8413 – DRG 532a	Explant and re-implant performed in the same
explantation and re-			hospitalization. DRG without CC was applied to
implantation (noninfection)			91.35% of SAEs that were not infections.
SAE requiring lead	16 826	€8413 * 2 – DRG 532a	Explant + new implant procedure. The DRG without
replacement (infection)			CC was applied in both hospitalizations for 8.65% of
			SAEs which were infections.
SAE requiring lead	8413	DRG 532a	Explant and re-implant performed in the same
replacement (noninfection)			hospitalization. DRG without CC was applied to
			91.35% of SAEs that were not infections.
SAE requiring surgery but	€2326	DRG 8a	
not device kit replacement			
SAE requiring	2077	DRG 35a	
hospitalization but not			
surgery			
Permanent device	8413	DRG 532a	Applied at the time of therapy discontinuation.
explantation			

^a^DRG Manual ICD-9-CM-2007 Version 24.0. Max National DRG Tariffs 2012.

^b^Annual National Report on Hospitalizations. https://www.salute.gov.it/portale/documentazione/p6_2_2_1.jsp?lingua=italiano&id=3277

DRG 7: PERIPH & CRANIAL NERVE & OTHER NERV SYST PROC W CC DRG 8: PERIPH & CRANIAL NERVE & OTHER NERV SYST PROC W/O CC

DRG 35: OTHER DISORDERS OF NERVOUS SYSTEM W/O CC DRG 531: SPINAL PROCEDURES W CC

DRG 532: SPINAL PROCEDURES W/O CC

Abbreviations: CC, complication or comorbidity; SAE, serious adverse event; SDO, hospital discharge records.

### Analyses Performed

To assess the cost impact of battery longevity of NR-SCS devices from the Italian NHS perspective, 2 scenario analyses were performed comparing Device C vs Device A and Device C vs Device B in both 1-step (DTI) and 2-step implant settings. The differentiating outputs that contributed to defining the different impacts of these devices were the mean cost per patient, survival time, number of SAEs, and number of device replacements.

## RESULTS

The results presented below are for both a DTI and a 2-step implant approach at 15 years. Under the model settings used, Device C (compared with Device A) was predicted to save an average of €4227 per patient over 15 years considering either a DTI or a 2-step approach. In both cases, the main part of the cost savings was due to the need for fewer device replacements for patients with Device C implants. **Table S1** and **Figure S1** show the main results and the cumulative costs over time, respectively, for the comparison of Device C and Device A.

Over 15 years, using a DTI approach, the undiscounted total costs for Device A vs Device C were €26 860 and €22 633, respectively: the confidence limits for mean costs per patient were €6327 to €38 939 for Device C and €9568 to €44 152 for Device A. For the 2-step implant, the costs were €25 982 for Device C and €30 240 for Device A: the confidence limits for mean costs per patient were €9774 to €42 189 for Device C and €12 939 to €47 542 for Device A (see **Figure S1**).

As shown in **Figure S1**, the steeper parts of the line appear when device replacements, the main cost drivers, occur. **Figure S1** also shows an increase of the mean costs over time, which is related to the ongoing costs of managing adverse events (which can occur at any time during the model horizon). Between Device C and Device A, there was a mean difference of 1.22 replacements over 15 years (0.97-2.19, respectively) in both the DTI and 2-step implant approaches, due to the different devices’ battery longevity.

**Table S2** and **Figure S2** show the main results and the cumulative costs over time, respectively, for the comparison of Device C and Device B. Device C was predicted to save an average of €2712 per patient (compared with Device B) over 15 years using either a DTI or a 2-step approach. Almost all of these savings were due to device replacements avoided with Device C. Using a DTI approach, the undiscounted total costs for Device B vs Device C were €25 111 and €22 399, respectively (confidence limits: Device C, €6317-€38 481; Device B, €8114-€42 109). For the 2-step implant, the costs were €25 809 for Device C and €28 521 for Device B (confidence limits: Device C, €9727-€41 891; Device B, €11 523-€45 519) (see **Figure S2**). Between Device C and Device B, there was a mean difference of 0.79 replacements over 15 years in both the DTI and 2-step implant approaches (0.91-1.70, respectively) due to the different device battery longevity.

Cost savings for Device C ranged from €3278 to €4898 vs Device A, and from €2461 to €4497 vs Device B, when device longevity was varied by 10% on either side of the mean. Considering a discount rate of 3% per year, the cost saving for Device C is confirmed in every scenario even with a slight variation of the absolute value. The main difference between the DTI and the 2-step implant approach, besides the specific device’s comparison, relates to the cost of the initial device and the second hospitalization costs.

## DISCUSSION

This economic analysis sought to estimate costs related to implantation and replacement of NR-SCS devices over a 15-year period from the Italian NHS perspective, using battery longevity data from 3 manufacturers and recording the expected incidence of SAEs, mean number of device replacements, and patient survival. We used Italian DRG tariffs to estimate the costs of device acquisition and implantation, device replacement, management of adverse events, and permanent withdrawal from therapy. Due to its increased battery longevity, Device C is expected to be less expensive than both Device A and Device B.

The results confirmed our hypothesis. Device C was demonstrated to save an average of €4227 per patient compared with Device A and €2712 per patient compared with Device B over 15 years using either a DTI or a 2-step approach. The overall conclusions did not change when device longevities were varied in sensitivity analysis. The calculated confidence intervals around the mean total cost per patient were relatively wide; this can be explained by using patient-level modeling, such that some patients will die earlier than others and thus have much lower total costs than those who survive until the end of the model horizon. The battery longevity of NR-SCS devices directly impacts long-term costs to a payer. The longer the device lasts, the fewer mean total cumulative costs the patient will incur, especially with regard to device replacement costs. With novel devices and specific programming settings, the lifetime cost per patient to a payer can be decreased. Furthermore, comparing the cost per patient over 15 years of Device C/DTI vs Device C/2-step approach in both different impulse settings, the DTI approach appears to be a cost-saving alternative for the NHS, saving approximately €3400.

A comparison of RC with NR device costs over 15 years would result in favor of RC devices in each setting. Nevertheless, because RC devices are not suitable for all patients, both RC and NR devices are currently available as therapeutical options. In recent years, there has been a rapid technological development in the features of NR devices, particularly regarding the extension of battery longevity, which has demonstrated an impact on resource consumption at the NHS system level. The analysis results represent a key element to support decision makers’ technological innovation choices, to reach patient pathway efficiency and optimization without compromising the patient’s safety and positive clinical outcome.

Moreover, the TRIAL-STIM study,^14^ a randomized controlled trial across 3 centers in the United Kingdom, has shown an absence of evidence supporting that an SCS screening trial strategy leads to better patient outcomes vs an approach without trial stimulation. Emergent themes favored the option for the DTI SCS procedure, including saving time for the patient (work absenteeism, in hospital, attending appointments), avoiding the worry for the patients about having “loose wires” in the 2-stage procedure, having only 1 period of recovery, and saving NHS resources.^14^

In line with this innovative approach, more relevance and importance should be attributed to patient-reported outcome measures, which should be included in every clinical study to provide a more complete picture of all the parameters that contribute to improving the quality of care from the patient’s perspective.

The findings of Chadwick et al^14^ indicate an overwhelming preference among participants for the DTI approach in the SCS procedure both before and after the implant, regardless of which procedure they had undergone. The qualitative study findings further support the TRIAL-STIM RCT results.^14^ The advantages of adopting new high-performance technology and the DTI approach appear to be clear from the NHS perspective. However, due to intrinsic characteristics of the Italian system, only 4 regions of 20 have a specific tariff (all inclusive) that better describes the DTI approach, even though the tariffs paid under this system appear insufficient to cover procedural plus device costs.

The goal of healthcare systems is to provide health, seeking to ensure the best outcomes for patients, constantly improving the quality of care, and increasing access to innovative therapies through the improvement of clinical appropriateness and the creation of organizational contexts that favor the uptake of innovation. In the provision of care, healthcare systems must maximize health outcomes for patients while optimizing costs. However, to date, healthcare systems are based on the delivery and financing of individual services in a disconnected way from their outcomes and value, often facing high and unsustainable costs and providing a quality of care that is not fully satisfying for patients.

To overcome the current challenges in delivering health care and to promote the transition to sustainable and quality healthcare systems, a paradigm shift is necessary that moves the focus from the volume of services to the patient’s health needs and the value of the care provided. It is necessary to focus on the healthcare systems’ value generated by the entire care pathway developed based on the patient’s needs, where value means the ratio between specific outcomes for a clinical condition and the cost necessary to achieve them.

Implementing this paradigm shift also means gradually adapting the financing systems, currently focused on individual services and in no way correlated to the outcomes produced by them, to payment models focused on the value produced by the care provided and therefore toward the realization of value-based health care. This analysis, even with its limitations, offers a way to evaluate and promote the value of innovation, taking into consideration the sustainability of the healthcare system.

The model needed several simplifying assumptions to be made to extrapolate the results to a long-term horizon and overcome a lack of empirical data for some inputs. Battery longevity could not translate into real-world clinical longevity as the programming of the device is highly personalized and various device programming and usage patterns could be used as a strategy in a real-world setting to prolong battery longevity.^7^

The model is likely to overestimate the incidence of SAEs in the long term because it was based on 2-year data from the French registry.^21^ However, this is a more conservative approach than limiting the occurrence of SAEs to the first 2 years of the model and provides a way of incorporating an elevated (but brief) increased risk of complications at the time of device replacement.

Management of SAEs was also largely based on data from the French registry that might have slight differences from Italian practice. General disease management costs were not included in the analysis as they were assumed to be the same for all device types (eg, pain medication, non-drug therapy). Nevertheless, assumptions were necessary regarding the need for explantation and replacement of device components. As a result, a significant proportion of SAEs were assumed to require lead and/or device replacement, which may overestimate SAE management costs in the long term. However, this effect is likely to be offset to some extent by the exclusion of nonserious events from the analysis. Furthermore, the model predicted a similar occurrence of SAEs across all manufacturers, so any differences in cost were minimal.

Additionally, the model assumed that, unless a patient withdrew from therapy, they would continue to receive device replacements according to the fixed time interval for their device (ie, no age limit was fixed beyond which a patient would not receive a replacement device). This assumption may therefore overestimate the average number of devices per patient, since a patient’s age is a factor in determining whether to replace an implantable device.

Device A and Device B have published longevity data. The nominal settings used from Proclaim™ clinician manual^17^ were 12 hours per day: 50 Hz frequency, 225 μs pulse width, and 5 mÅ amplitude at 500 Ω impedance compared with flagship model 3660. The setting from Boston Scientific’s Alpha IFU,18 programmed at 4.1 mÅ, 280 µs, 40 Hz, 1 area, 730 Ω, 2 contacts, were used directly in the model. Additionally, the stimulation settings for these 2 devices, which were used to derive the expected battery longevity for Device C, are publicly available. Thus, the expected longevity of Device C was not based on real-world observational data of patients implanted with the device. However, using equivalent stimulation settings to the competitor devices did ensure that a like-for-like comparison was made in each case. Using a fixed device replacement interval for each device also ignores the fact that device failure follows a distribution in which some devices would last less than the mean longevity, and some would last longer (due to variation in patient stimulation settings).

However, a fixed interval approach was used to simplify the model and allow the mean longevity to be easily changed; this would not be possible with a time-dependent distribution approach.

## CONCLUSIONS

Extended SCS battery longevity can be translated into tangible cost savings for payers. Furthermore, a DTI approach for SCS supports NHS cost efficiencies and can offer the additional benefits of optimizing operating room time and having only 1 recovery period for the patient.

Further studies, ideally multicountry, large, randomized comparisons of different technologies, including real-world data from Device C, are needed to statistically demonstrate the reduction in complication rate associated with the DTI approach and better inform shared decision-making about the potential costs and benefits for future investments. Furthermore, the institution of an Italian National Registry could cover the lack of data in some areas and offer the opportunity to run the model with customized real-world evidence.

### Disclosures

The author(s) declared the following potential competing interest with respect to the research, authorship, and/or publication of this article: the manuscript was developed by F.T., S.E., E.T., F.B. and M.C. from the health economics department of Medtronic, with the collaboration of G.S. and G.P., from the Pain Unit of Santa Maria Maddalena Hospital–Advanced Algology Research, who declare no competing interests.

## Supplementary Material

Online Supplementary Material
