# Supplementary material for: Optimizing Healthcare Expenditure for Spinal Cord Stimulation in Italy: The Value of Battery Longevity Improvement and a Direct-to-Implant Approach
Source: J Health Econ Outcomes Res. 2024 May 28;11(1):149–56. doi: 10.36469/001c.116177 (PMC11139019; doi:10.36469/001c.116177)
Supplement: Online Supplementary Material [file jheor_2024_11_1_116177_229419.pdf]

Journal of Health Economics and Outcomes Research

Online Supplementary Material

Optimizing Healthcare Expenditure for Spinal Cord Stimulation in Italy: The Value of Battery Longevity Improvement and a Direct-to-Implant Approach. *JHEOR*. 2024;11(1):149-156. doi:10.36469/jheor.2024.116177

Table S1: Vanta™ PC (Medtronic) vs Proclaim™ XR PC (Abbott)

Figure S1: Cumulative Costs of Vanta™ PC (Medtronic) vs Proclaim™ XR PC (Abbott)

Table S2: Vanta™ PC (Medtronic) vs Alpha™ PC (Boston Scientific)

Figure S2: Cumulative costs of Vanta™ PC (Medtronic) vs. Alpha™ PC (Boston Scientific)

This supplementary material has been provided by the authors to give readers additional information about their work.

This is an open-access article distributed under the terms of the Creative Commons Attribution 4.0 International License (CCBY-4.0). View this license's legal deed at http://creativecommons.org/licenses/by/4.0 and legal code at http://creativecommons.org/licenses/by/4.0/legalcode for more information.

Table S1. Vanta™ PC (Medtronic) vs Proclaim™ XR PC (Abbott)

**Outcome**	**Direct-to-Implant**			**2-Step Implant**		
	**Vanta™ PC**	**Proclaim ™ XR PC**	**Savings**	**Vanta™ PC**	**Proclaim™ XR PC**	**Savings**
Mean total cost (undiscounted) (€)	22 633	26 860	-4227	25 982	30 240	-4259
Initial device and implant (€)	8973	8 73	0	12 383	12 383	0
Battery replacements (€)	2136	6326	-4190	2141	6289	-4148
Adverse events (€)	11 524	11 560	-36	11 457	11 568	-111
Lower confidence limit for mean cost per patient (€)	6327	9568	-3240	9774	12 939	-3165
Upper confidence limit for mean costs per patient (€)	38 939	44 152	-5214	42 189	47 542	-5352
Mean patient survival time (years)	14.5	14.5	0.0	14.5	14.5	0.0
Mean No. of serious adverse events	1.426	1.438	-0.012	1.422	1.433	-0.011
Mean No. of device replacements	0.97	2.20	-1.23	0.97	2.19	-1.22
% of patients withdrawing	45	45	0	45	45	0

Figure S1. Cumulative Costs of Vanta™ PC (Medtronic) vs Proclaim™ XR PC (Abbott)

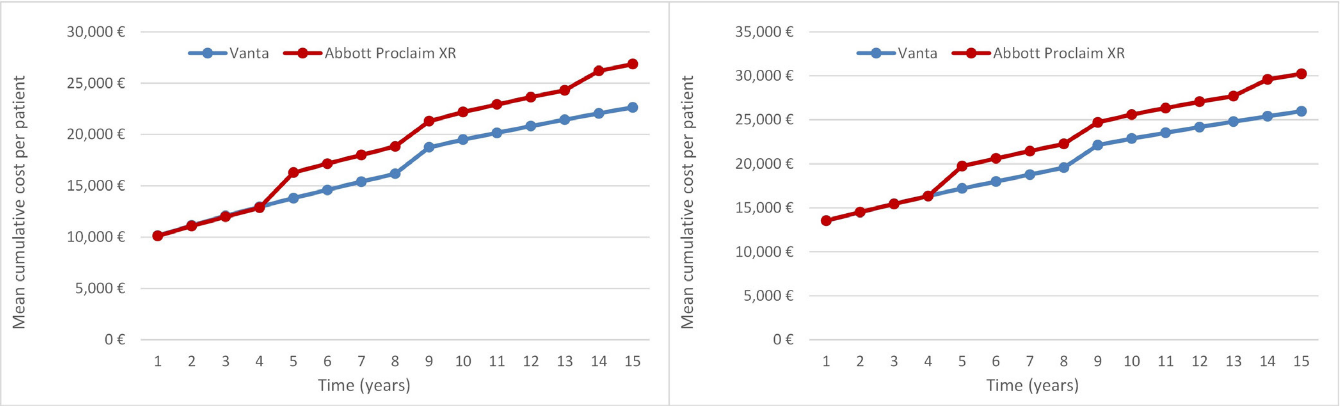

Left: direct-to-implant; *right*, 2-step approach.

Table S2. Vanta™ PC (Medtronic) vs Alpha™ PC (Boston Scientific)

Outcome	Direct-to-Implant			2-Step Implant		
	**Vanta™ PC**	**Alpha™ PC**	**Savings**	**Vanta™ PC**	**Alpha™ PC**	**Savings**
Mean total cost (undiscounted) (€)	22 399	25 111	-2712	25 809	28 521	-2712
Initial device and implant (€)	8973	8973	0	12 383	12 383	0
Battery replacements (€)	1940	4623	-2682	1940	4623	-2682
Adverse events (€)	11 486	11 515	-30	11 486	11 515	-30
Lower confidence limit for mean cost per patient (€)	6317	8114	-1796	9727	11 523	-1796
Upper confidence limit for mean costs per patient (€)	38 481	42 109	-3628	41 891	45 519	-3628
Mean patient survival time (years)	14.5	14.5	0.0	14.5	14.5	0.0
Mean No. of serious adverse events	1.420	1.424	-0.004	1.420	1.424	-0.004
Mean No. of device replacements	0.91	1.70	-0.79	0.91	1.70	-0.79
% of patients withdrawing	45%	45%	0%	45%	45%	0%

Figure S2. Cumulative Costs of Vanta™ PC (Medtronic) vs Alpha™ PC (Boston Scientific)

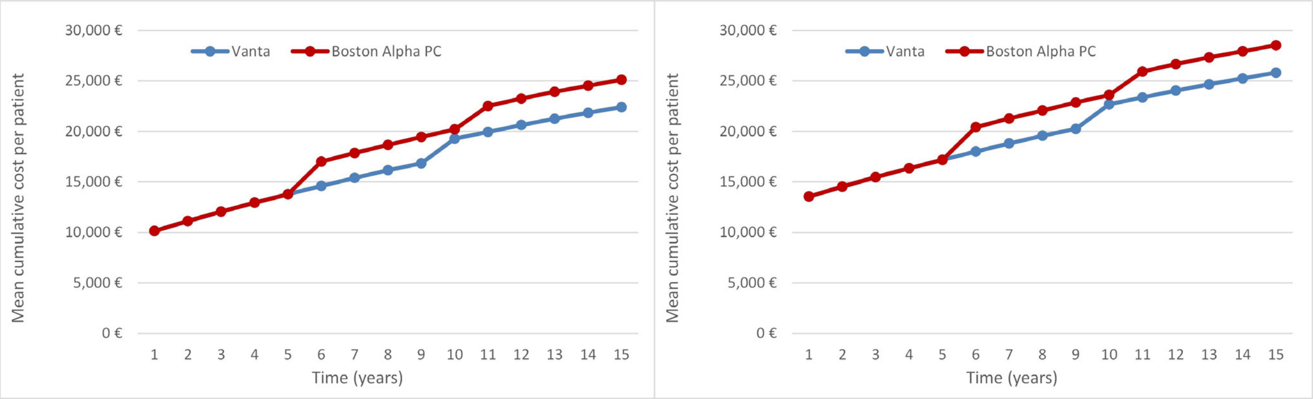

Left: direct-to-implant; *right*, 2-step approach.
